# Untargeted metabolomic approach to study the serum metabolites in women with polycystic ovary syndrome

**DOI:** 10.1186/s12920-021-01058-y

**Published:** 2021-08-20

**Authors:** Ying Yu, Panli Tan, Zhenchao Zhuang, Zhejiong Wang, Linchao Zhu, Ruyi Qiu, Huaxi Xu

**Affiliations:** 1 Institute of Laboratory Medicine, Jiangsu Key Laboratory of Laboratory Medicine, Jiangsu University, Zhenjiang, 210013 Jiangsu People’s Republic of China; 2 Department of Laboratory Medicine, Chinese Medicine Hospital of Zhejiang, Hangzhou, 310006 Zhejiang People’s Republic of China

**Keywords:** Metabonomics analysis, UPLC-HRMS, Polycystic ovary syndrome, Serum metabolites

## Abstract

**Background:**

Polycystic ovary syndrome (PCOS) is not only a kind of common endocrine syndrome but also a metabolic disorder, which harms the reproductive system and the whole body metabolism of the PCOS patients worldwide. In this study, we aimed to investigate the differences in serum metabolic profiles of the patients with PCOS compared to the healthy controls.

**Material and methods:**

31 PCOS patients and 31 matched healthy female controls were recruited in this study, the clinical characteristics data were recorded, the laboratory biochemical data were detected. Then, we utilized the metabolomics approach by UPLC-HRMS technology to study the serum metabolic changes between PCOS and controls.

**Results:**

The metabolomics analysis showed that there were 68 downregulated and 78 upregulated metabolites in PCOS patients serum compared to those in the controls. These metabolites mainly belong to triacylglycerols, glycerophosphocholines, acylcarnitines, diacylglycerols, peptides, amino acids, glycerophosphoethanolamines and fatty acid. Pathway analysis showed that these metabolites were enriched in pathways including glycerophospholipid metabolism, fatty acid degradation, fatty acid biosynthesis, ether lipid metabolism, etc. Diagnosis value assessed by ROC analysis showed that the changed metabolites, including Leu–Ala/Ile–Ala, 3-(4-Hydroxyphenyl) propionic acid, Ile–Val/Leu–Val, Gly–Val/Val–Gly, aspartic acid, DG(34:2)_DG(16:0/18:2), DG(34:1)_DG(16:0/18:1), Phe–Trp, DG(36:1)_DG(18:0/18:1), Leu–Leu/Leu–Ile, had higher AUC values, indicated a significant role in PCOS.

**Conclusion:**

The present study characterized the difference of serum metabolites and related pathway profiles in PCOS patients, this finding hopes to provide potential metabolic markers for the prognosis and diagnosis of this disease.

## Background

Around the world, approximately 15–20% of the childbearing age women are affected with polycystic ovary syndrome (PCOS) according to the Rotterdam criteria [1]. PCOS is not only one of the most common endocrine syndrome but also a metabolic disorder, which is mainly characterized by hyperandrogenism (HA) and insulin resistance (IR). The main clinical manifestations of PCOS patients are menstrual cycle irregular, oligo-ovulation, polycystic ovarian morphology, IR induced obesity, HA induced hirsutism and acne [2]. But the diagnosis of PCOS remains a controversial issue and the criteria are continue to be updated [3–5]. Except for the impairment of ovarian function and the overall body metabolism, the thereby caused anovulatory infertility and recurrent pregnancy loss also have tremendous harm to PCOS patients. In addition, due to the dysfunction of the ovary and metabolism, the incidence of negative consequences, such as gynecological cancer, hypertension, atherosclerosis, type 2 diabetes mellitus (T2DM), and cardiovascular disease (CVD), also seem to be higher in PCOS women than those in normal populations [6–8]. In light of these risks, there is a strong need of reliable biochemical or molecular markers, which would enable to make the accurate diagnosis and effective therapy of PCOS.

Yet, the knowledge of the mechanisms underlying PCOS pathophysiology is still insufficient, and this restricts the development of available or effective therapies to ameliorate the symptoms of PCOS or related metabolic complications [9]. And shockingly, half of all women with PCOS are thought to remain undiagnosed. Genomic, proteomic, and metabolomic approaches to study the pathogenesis of diseases have been introduced to various diseases researching. Metabolomics involves the comprehensive characterization of metabolites in biological systems, and is widely applied for better disease diagnosis, understanding the potential mechanisms, identifying novel drug targets, customizing drug treatments and monitoring therapeutic outcomes [10]. The untargeted metabolomic approach, known as metabolic fingerprinting, mainly focuses on the identification and quantification of as many as possible low-molecular-weight compounds present in tested samples. This approach is commonly applied to uncover metabolic profiles, metabolic markers and to reveal new insights into the mechanisms underlying the pathogenesis of human diseases, including PCOS [11].

In this study, based on metabolomics approach by utilizing an ultra-performance liquid chromatography–high resolution mass spectrometry (UPLC-HRMS) technology, we aim to characterize the metabolic fingerprints of the PCOS patients, in hope of identifying potential metabolic marker for the prognosis and diagnosis of this disease.

## Material and methods

### Study subjects

All of the PCOS patients and healthy controls were recruited from the Zhejiang Provincial Hospital of Chinese Medicine (Hangzhou, China). This study was approved by the Ethics Committee of Zhejiang Provincial Hospital of Chinese Medicine. The signed informed consents were obtained from all the participators before inclusion in this study.

According to the Rotterdam criteria, 2003, PCOS patients can be diagnosed if two of the three criteria are present after excluding congenital adrenal hyperplasia, Cushing’s syndrome, androgen secreting tumors, or other related disorders. The three criteria are (1) oligo- and/or anovulation; (2) clinical and/or biochemical signs of HA (clinical manifestations of HA include the presence of acne, hirsutism, and androgenic alopecia); (3) polycystic ovaries by ultrasound examination: the presence of 12 or more follicles in each ovary measuring 2–9 mm in diameter and/or ovarian volume > 10 cm^3^.

The inclusion criteria for PCOS cases in this study were: diagnosed with PCOS according to the Rotterdam criteria, 2003 [4]; adolescent females (18–40 years old); had at least 2 years of menstrual history. Exclusion criteria: had received any androgenic drug or sex steroid therapy in the past 3 months before the study; current pregnant, delivery or miscarriage within the preceding 3 months; congenital adrenal hyperplasia, androgen-secreting tumors, and other diseases with HA, thyroid dysfunction, hyperprolactinemia, cardiovascular diseases, diabetes or any chronic diseases. The control group were healthy female volunteers: 18–40 years old, regular menstrual cycles and normal androgen levels, without PCOS and IR, and no evident disease was detected during the study. According to the above-mentioned inclusion/exclusion criteria, a total of 31 PCOS patients and 31 healthy participants were included from December of 2018 to April of 2019 in the present study.

The clinical characteristics data of the enrolled participators were recorded at the time of recruitment. After fasting for 8 h, the blood sample from each participator was collected. The serum samples were stored at − 80 ℃ for subsequent assay.

### Clinical laboratory tests

Serum concentrations of fasting glucose, fasting insulin, follicle-stimulating hormone (FSH), luteinizing hormone (LH), estradiol (E2), prolactin (PRL), testosterone (T), progesterone (P), total cholesterol (TC), triglyceride (TG), high-density lipoprotein cholesterol (HDL-c), low-density lipoprotein cholesterol (LDL-c) in all PCOS patients and control participants were detected by Immulite 2000 analyzer (Siemens Healthcare Diagnostics Products Ltd., UK) using two-site chemiluminescent immunometric assays.

### Sample preparation and metabolite extraction

*The polar metabolome extraction*: After thawed at 4 ℃, a 100 μL serum samples were added with 400 μL methanol–acetonitrile (1:1, v:v; including isotope internal standard tryptophan -d5, cetylic acid-[13C]12), centrifugated at 15,000 g for 15 min. Then a 200 μL supernatants were dried under low-temperature vacuum (Thermo Scientific, USA) to obtained the sample for UPLC-HRMS analysis. Before analysis, the samples were redissolved with 100 μL 10% methanol (including multiple internal standards).

*The lipidomic metabolome extraction:* After thawed at 4 ℃, a 50 μL serum samples were added with 300 μL methanol (including internal standards: Ceramide (d18:1/17:0), PC(17:0/17:0), TG(15:0/15:0/15:0)), swirled for 120 s, and added with 900 μL MTBE, 250 μL ultrapure water. After the vortex was mixed and vibrated at room temperature for 15 min, the solution was placed under 4 ℃, 30 min for stratifying. Then 900 μL supernatants were transferred into EP pipe and dried under low-temperature vacuum (Thermo Scientific, USA) to obtain the sample for UPLC-HRMS analysis. Before analysis, the samples were redissolved with a 600 μL acetonitrile–isopropanol mixture.

### UPLC-HRMS instrumentation and measurement conditions

Untargeted metabolomics analysis was conducted by using three different analytical methods (M1-3) on an Ultimate 3000 ultra-high performance liquid chromatography coupled with Q ExactiveTM quadrupole-Orbitrap high-resolution mass spectrometer (UPLC-HRMS) system (Thermo Scientific, USA).

#### UPLC system

Untargeted metabolomics analysis was conducted by using three different analytical methods (M1-3). Method 1 and 2 (M1, M2) were used for the polar metabolome extracts analysis on the UPLC-HRMS system with positive and negative ionization detection, respectively. Metabolites were separated by an AcquityTM HSS C18 column (Waters Co., USA, 2.1 × 100 mm) for M1, and eluted by 0.1% formate/water (A) and acetonitrile (B) in a linear gradient from 2% organic mobile phase to 98% in 10 min. Furthermore, other mobile phases consisting of water and ammonium acetonitrile/methanol both containing ammonium bicarbonate buffer salt were employed to eluted metabolites separated on an AcquityTM BEH C18 column (Waters Co., USA, 1.7 μm, 2.1 × 100 mm), the gradient was used as follow: from 0–10 min, 2% organic phase ramped to 100%, and from 10 to 15 min, column washing and equilibrating. Untargeted lipidomic analysis was operated based on Method 3 (M3), the chromatographic separation conditions were maintained under positive and negative ionization detection mode, respectively. The used column was an Accucore C30 core–shell column, the mobile phase was 60% acetonitrile in water (A) and 10% acetonitrile in isopropanol (B) both containing 10 mM ammonium formate and 0.1% formate. The separation gradient was optimized as follows: initial 10% B, ramping to 50% in 5 min, and further increasing to 100% in 23 min, then the rest 7 min for column washing and equilibration. For Method 1–3, the flow rate was 0.4 mL/min, injection volume was 5 μL, and the column temperature was 50 ℃.

#### Mass spectrometer system

For Method 1–2, the quadrupole-Orbitrap mass spectrometer was all operated under identical ionization parameters with a heated electrospray ionization source except ionization voltage including sheath gas 45 arb, aux gas 10 arb, heater temperature 355 ℃, capillary temperature 320 ℃ and S-Lens RF level 55%. The metabolome extracts were profiled with full scan mode under 70,000 FWHM resolution with AGC 1E6 and 200 ms max injection time. The scan range was 70–1000 m/z. QC samples were repeatedly injected to acquired Top 10 data-dependent MS2 spectra (full scan-ddMS2) for comprehensive metabolite and lipid structural annotation. 17,500 FWHM resolution settings were used for full MS/MS data acquisition. Apex trigger, dynamic exclusion, and isotope exclusion were turned on, precursor isolation window was set at 1.0 Da. Stepped normalized collision energy was employed for collision-induced disassociation of metabolite using ultra-pure nitrogen as fragmentation gas. All the data acquired in centroid format. For Method 3, the ionized lipid molecules were detected using the same parameters as the previous description 6.3.1. 300–2000 m/z lipid extracts were profiled with the same parameters as the metabolome used. Lipid was structurally identified through acquiring data-dependent MS2 spectra, the key settings included 70,000 FWHM full scan resolution, 17,500 FWHM MS/MS resolution, loop count 10, AGC target 3e6, maximum injection time 200 ms and 80 ms for full scan, and MS/MS respectively, dynamic exclusion 8 s. Stepped normalized collision energy 25% + 40% and 35% were employed for positive and negative mode after optimization.

### Metabolomics data analysis

The full scan and data-dependent MS2 metabolic profiles data were further processed with Compound Discoverer software for comprehensive component extraction. The polar metabolites were structurally annotated through searching acquired MS2 against a local proprietary iPhenomeTM SMOL high-resolution MS/MS spectrum library created using authentic standards, NIST 17 Tandem MS/MS library (National Institute of Standards and Technology), local version MoNA (MassBank of North America), as well as mzCloud library (Thermo Scientific, USA). Besides, the exact m/z of MS1 spectra was searched against a local KEGG, HMDB metabolite chemical database. For metabolite identification or structural annotation, mass accuracy of precursor within ± 5 ppm was a prerequisite, meanwhile, isotopic information including at least 1 isotopes within 10 ppm and a fit score of relative isotopic abundance pattern 70% were introduced to confirm the chemical formula in addition to exact mass. Furthermore, retention time information as well as high-resolution MS/MS spectra similarity was employed to strictly confirm the structural annotation of metabolites. The area under curve (AUC) values as extracted as quantitative information of metabolites with XCalibur Quan Browser information, all peak areas data for the annotated metabolites were exported into Excel software for trim and organization before statistics (Microsoft, USA). And on the other hand, untargeted lipidomics data was processed with LipidSearch software including peak picking, lipid identification. The acquired MS2 spectra were searching against in silico predicted spectra of a diverse phospholipid, neutral glycerolipid, sphingolipid, neutral glycosphingolipids, glycosphingolipids, steroids, fatty ester, etc. The mass accuracy for precursor and MS/MS product ions searching were 5 ppm and 5 mDa, respectively. The MS/MS similarity score threshold was set at 5. The potential ionization adduct including hydrogen, sodium, ammonium for positive and hydrogen loss, formate and acetate adduct for negative mode. The lipid identification was strictly manually checked and investigated one by one to eliminate false positives chiefly basing on peak shake, adduct ions behavior, fragmentation pattern, and chromatographic behavior.

### Statistical analysis

All the clinical data were computed using SPSS18.0 version software. An unpaired, two-tailed Student *t* test was performed on clinical biochemical data, the chi-square test was used for comparison of categorical variables. *p* value < 0.05 was considered to be statistically significant. The metabolome and lipidome data deriving from different measurements were normalized to sample weight used before further process, respectively. Then, the resultant quantitative information from the foregoing methods was merged and those detected with multiple methods were excluded to guaranteed uniqueness of metabolite and lipid, and then Log10 transformed for final statistical analysis. The principal component analysis was conducted with SIMCA-P software (Umetrics, Sweden), and another univariate analyses including independent sample t-test and *p* value FDR adjust, as well as metabolic pathway analysis was conduct on the MetaboAnalyst website.

## Results

### Clinical characteristics and biochemical data of the study subjects

The Clinical characteristics and biochemical data of the study subjects were collected and analyzed (Table 1). In this study, the study subjects included 31 healthy controls and 31 PCOS women. There are no statistical differences for the age, BMI between the two groups (*p* value > 0.05). For biochemical data, the levels of fasting glucose, LH, T, TG, LDL-c, and LH/FSH ratio were significantly higher in PCOS patients than those in controls, the levels of PRL, HDL-c were significantly lower in PCOS patients than those in controls (*p* value < 0.05).

**Table 1:** Clinical characteristic and biochemical data of the study subjects

	Control (n = 31)	PCOS (n = 31)	*p* value
Age [years]	24.52 ± 2.31	24.20 ± 4.49	0.750
BMI [kg/m^2^]	20.48 ± 2.67	22.27 ± 3.56	0.081
Fasting glucose [mmol/L]	4.68 ± 0.42	5.25 ± 1.20	0.026 < 0.05
Fasting insulin [pmol/L]	6.9 ± 3.38	14.10 ± 13.83	0.007 < 0.05
FSH [IU/L]	5.15 ± 1.32	5.59 ± 2.86	0.440
LH [IU/L]	5.85 ± 2.74	9.82 ± 8.57	0.017 < 0.05
LH/FSH	1.14 ± 0.50	1.73 ± 0.97	0.003 < 0.01
PRL [mIU/L]	466.83 ± 231.05	309.64 ± 158.59	0.003 < 0.01
E2 [pmol/L]	209.95 ± 127.20	220.25 ± 246.80	0.837
T [nmol/L]	1.12 ± 0.40	1.65 ± 0.67	< 0.001
P [nmol/L]	0.93 ± 0.36	1.05 ± 0.80	0.451
TC [mmol/L]	4.44 ± 0.63	4.65 ± 0.76	0.306
TG [mmol/L]	0.75 ± 0.29	1.13 ± 0.51	0.002 < 0.05
HDL-c [mmol/L]	2.23 ± 0.52	1.46 ± 0.41	< 0.001
LDL-c [mmol/L]	1.69 ± 0.29	2.54 ± 0.62	< 0.001

*BMI* body mass index, *FSH* follicle-stimulating hormone, *LH* luteinizing hormone, *PRL* prolactin, *E2* estradiol, *T* testosterone, *P* progesterone, *TC* total cholesterol, *TG* triglyceride, *HDL-c* high-density lipoprotein cholesterol, *LDL-c* low-density lipoprotein cholesterol

### Multivariate statistical analysis

**Fig. 1:**
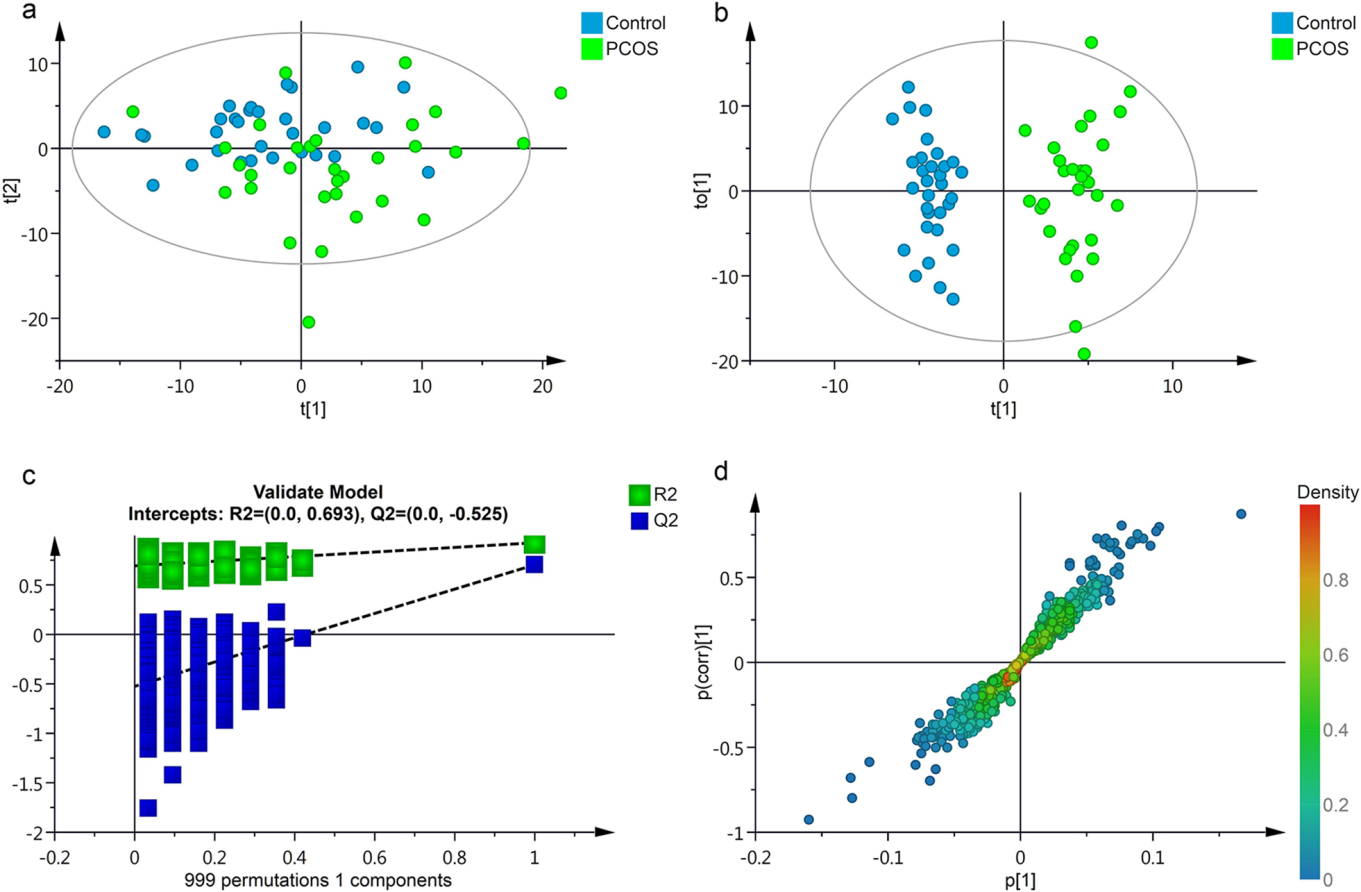
Multivariate statistical analysis of the serum metabolites in the study subjects. **a** unsupervised PCA score plots of metabolic phenotypes between PCOS and CON groups. Metabolomics data were log-transformed and scaled to unit variance for modeling. Model parameter: R^2^X = 0.86 (cumulative variance proportion of 9 principal components). **b** Score plot of OPLS-DA modeling to maximize inter-group differentiation of metabolomic data between PCOS and CON groups. Model parameter: 1 predictive component + 2 orthogonal component, R^2^Y = 0.93, Q^2^ = 0.70. **c** 999 times permutation test result of OPLS-DA modeling. **d** S-plot of OPLS-DA modeling

The PCA (principal component analysis) analysis outlined the original distribution of metabolites in PCOS and control subjects. As shown in Fig. 1a, the score plot of PCA suggested that there are no obvious outlier samples in the two groups. The scatter plot classification in PCOS and control groups was observed in t[2] axis, but failed to separate in t[1] axis. Hence, a POLS-DA model was applied for further analysis. As the results indicated in Fig. 1b, PCOS samples could be clearly distinguished from the healthy control samples. The models possessed a satisfactory fit of R^2^ = 0.93, Q^2^ = 0.70, which indicated the significant discrimination of the serum metabolomics signature between the control and PCOS groups. In Fig. 1c, permutation plots of the OPLS-DA model repeated 999 times verified the reliability of the model. S plot of the OPLS-DA model indicated the influence of metabolite expression level on metabolic phenotype classification (Fig. 1d).

### Significant changed metabolotics identification by UPLC-HRMS

**Fig. 2:**
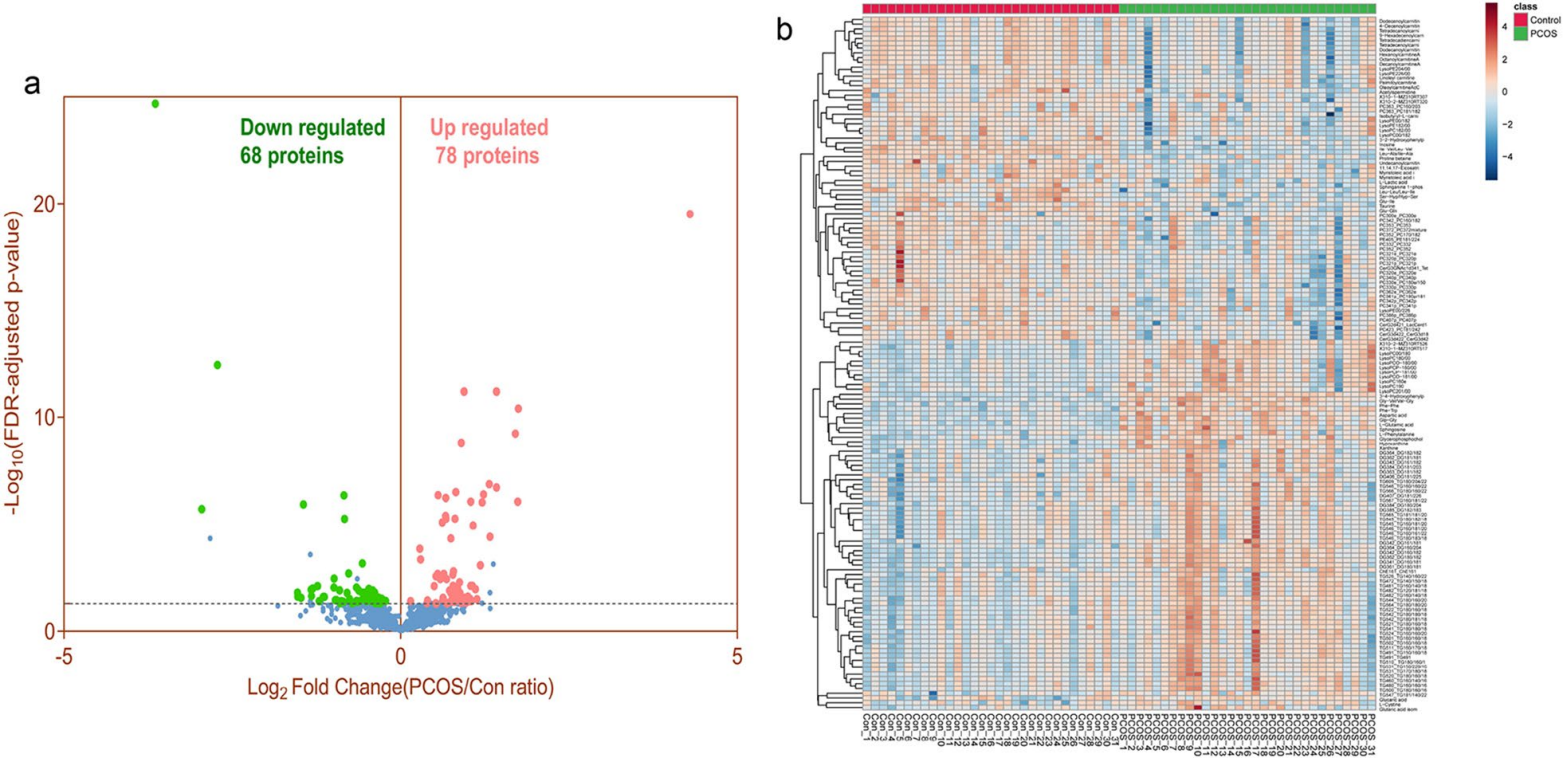
Identification of significant metabolotics by UPLC-HRMS. **a** Volcano plot to visualize differential metabolites of significance between PCOS and CON group. Metabolites with FDR adjusted *p* value ≤ 0.05 were highlighted with red (up-regulated) and green color (down-regulated), respectively. **b** Clustering analysis result using differential metabolites of significance in Student’s t-test analysis (FDR adjusted *p* value < 0.05)

**Fig. 3:**
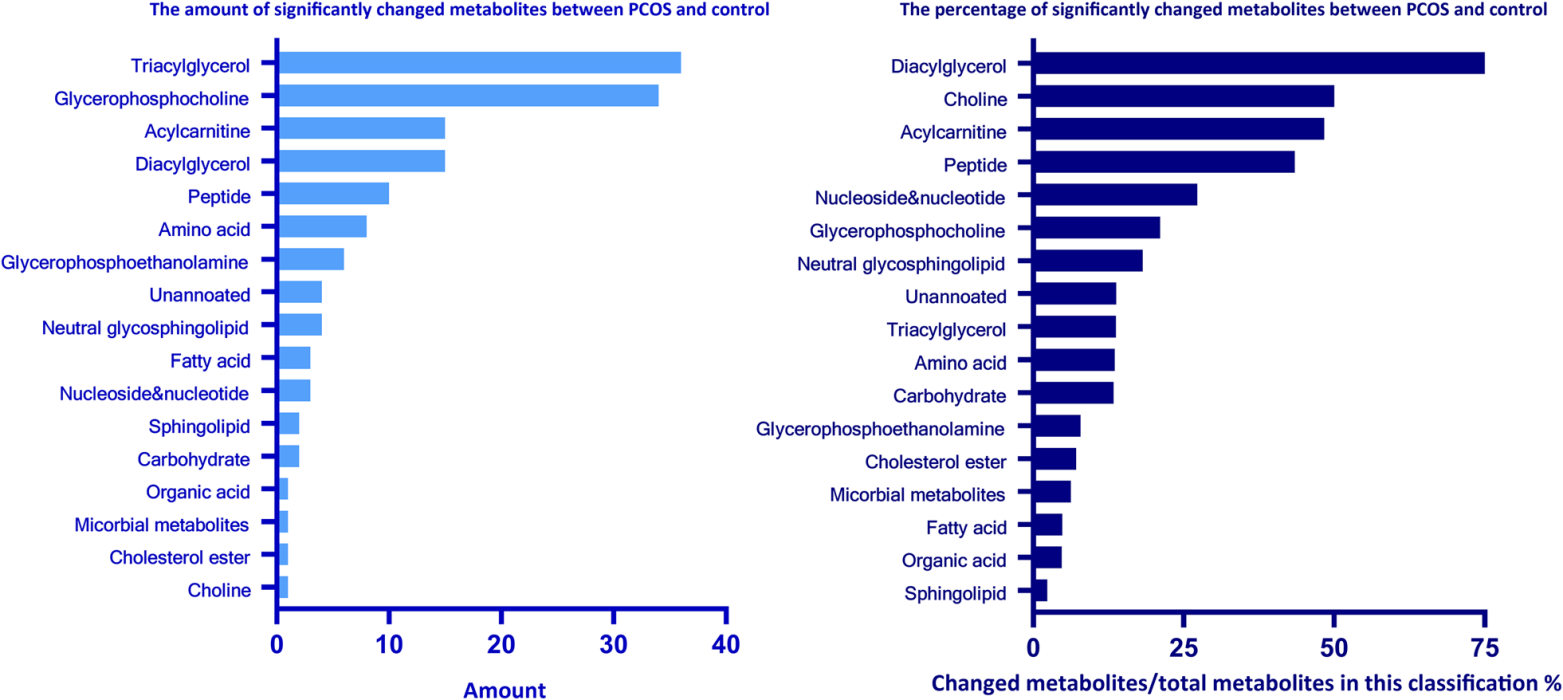
Chemical structure classification of differential metabolites between PCOS and control group

The variables with FDR adjusted *p* value < 0.05 were selected as remarkable significance in the OPLS-DA model. As a result, a total of 146 significantly changed metabolites were identified and selected as potential biomarkers of PCOS for subsequent analysis. The volcano plot showed that compared to the control group, among these metabolites, 68 were downregulated, 78 were upregulated (Fig. 2a, Table 2). Heatmap of these 146 significantly changed metabolites in 31 PCOS samples and 31 control samples indicated that these metabolites have clustering correlativity in PCOS patients from healthy controls (Fig. 2b). After chemical structure classification of the identified 146 differential metabolites, Fig. 3 showed that the significantly changed metabolites mainly belongs to the classes of triacylglycerol (36 metabolites), glycerophosphocholine (34 metabolites), acylcarnitine (15metabolites), diacylglycerol (15 metabolites), peptide (10 metabolites), amino acid (8 metabolites), glycerophosphoethanolamine (6 metabolites), fatty acid (FA) (3 metabolites), etc. And the classifications of top percentage of significantly changed metabolites (changed metabolites/total metabolites in this classification) were diacylglycerol (78.95%), choline (50.00%), acylcarnitine (48.39%), Peptide (43.48%), nucleoside & nucleotide (27.27%), glycerophosphocholine (21.12%), neutral glycosphingolipid (18.18%), triacylglycerol (13.74%), etc.

**Table 2:** The details about the differential metabolites identified in PCOS patients

Class	Metabolite	HMDB ID	Detection method	Mass accuracy (PPM)	FDR adjusted *p* value	Fold change PCOS/Con
Acylcarnitine	Palmitoylcarnitine(AcCa(16:0)	HMDB0000222	M1	− 1.54	0.0386	0.742
Acylcarnitine	Oleoylcarnitine(AcCa(18:1)	HMDB0005065	M1	− 2.08	0.0019	0.585
Acylcarnitine	Undecanoylcarnitine(AcCa(11:0)	HMDB0013321	M1	− 1.68	0.0000	0.561
Acylcarnitine	Isobutyryl-l-carnitine(AcCa(4:0)	HMDB0000736	M1	− 0.47	0.0384	0.543
Acylcarnitine	Tetradecanoylcarnitine(AcCa(14:0)	HMDB0005066	M1	− 1.53	0.0120	0.534
Acylcarnitine	Linoleyl carnitine(AcCa(18:2)	HMDB0006469	M1	− 2.03	0.0034	0.504
Acylcarnitine	Dodecenoylcarnitine(AcCa(12:1)	HMDB0013326	M1	− 1.62	0.0242	0.456
Acylcarnitine	4-DecenoylcarnitineAcCa(10:1)	HMDB0013205	M1	− 1.67	0.0242	0.451
Acylcarnitine	Hexanoylcarnitine(AcCa(6:0)	HMDB0000705	M1	− 0.55	0.0386	0.432
Acylcarnitine	9-Hexadecenoylcarnitine(AcCa(16:1)	HMDB0013207	M1	− 1.33	0.0076	0.425
Acylcarnitine	Tetradecenoylcarnitine(AcCa(14:1)	HMDB0002014	M1	− 1.30	0.0218	0.399
Acylcarnitine	Dodecanoylcarnitine(AcCa(12:0)	HMDB0002250	M1	− 1.50	0.0112	0.398
Acylcarnitine	Octanoylcarnitine(AcCa(8:0)	HMDB0000791	M1	− 1.52	0.0271	0.359
Acylcarnitine	Tetradecadiencarnitine(AcCa(14:2)	HMDB0013331	M1	− 1.49	0.0239	0.346
Acylcarnitine	Decanoylcarnitine(AcCa(10:0)	HMDB0000651	M1	− 1.09	0.0152	0.346
Amino acid	Aspartic acid	HMDB0000191	M2	3.33	0.0000	1.920
Amino acid	l-Glutamic acid	HMDB0000148	M2	3.78	0.0000	1.764
Amino acid	l-Cystine	HMDB0000192	M2	1.79	0.0356	1.319
Amino acid	l-Phenylalanine	HMDB0000159	M1	− 0.20	0.0004	1.228
Amino acid	Acetylspermidine	HMDB0001276	M1	− 0.92	0.0300	0.786
Amino acid	Taurine	HMDB0000251	M2	3.78	0.0158	0.674
Amino acid	3-(2-Hydroxyphenyl)propanoic acid	HMDB0033752	M2	3.36	0.0413	0.555
Amino acid	Proline betaine	HMDB0004827	M1	− 0.08	0.0098	0.405
Carbohydrate	Glucaric acid	HMDB0000663	M2	2.69	0.0459	1.961
Carbohydrate	l-Lactic acid	HMDB0000190	M2	3.88	0.0299	0.822
Cholesterol ester	ChE(16:1)_ChE(16:1)	HMDB0000658	M3	− 0.36	0.0475	1.435
Choline	Glycerophosphocholine	HMDB0000086	M1	− 0.92	0.0000	2.109
Diacylglycerol	DG(34:2)_DG(16:0/18:2)	HMDB0007103	M3	2.54	0.0000	3.358
Diacylglycerol	DG(36:4)_DG(16:0/20:4)	HMDB0007113	M3	2.35	0.0000	3.338
Diacylglycerol	DG(34:1)_DG(16:0/18:1)	HMDB0007101	M3	3.49	0.0000	3.260
Diacylglycerol	DG(36:1)_DG(18:0/18:1)	HMDB0007159	M3	2.88	0.0000	2.683
Diacylglycerol	DG(34:3)_DG(16:1/18:2)	HMDB0007132	M3	0.49	0.0000	2.509
Diacylglycerol	DG(36:2)_DG(18:0/18:2)	HMDB0007161	M3	1.49	0.0000	2.491
Diacylglycerol	DG(34:2)_DG(16:1/18:1)	HMDB0007131	M3	2.14	0.0008	2.271
Diacylglycerol	DG(40:7)_DG(18:1/22:6)	HMDB0007208	M3	− 0.89	0.0300	2.043
Diacylglycerol	DG(38:4)_DG(18:1/20:3)	HMDB0007198	M3	1.90	0.0051	1.835
Diacylglycerol	DG(36:3)_DG(18:1/18:2)	HMDB0007219	M3	2.40	0.0015	1.722
Diacylglycerol	DG(36:4)_DG(18:2/18:2)	HMDB0007248	M3	− 0.73	0.0075	1.716
Diacylglycerol	DG(36:2)_DG(18:1/18:1)	HMDB0007218	M3	3.21	0.0022	1.711
Diacylglycerol	DG(40:6)_DG(18:1/22:5)	HMDB0007207	M3	1.92	0.0493	1.610
Diacylglycerol	DG(38:5)_DG(18:2/18:3)	HMDB0007112	M3	1.76	0.0036	1.606
Diacylglycerol	DG(38:4)_DG(18:0/20:4)	HMDB0007170	M3	2.64	0.0020	1.560
Fatty acid	Dihomo-alpha-linolenic acid (FFA(20:3n3)	HMDB0060039	M2	1.69	0.0386	0.704
Fatty acid	Myristoleic acid isomer2	HMDB0002000	M2	2.46	0.0343	0.517
Fatty acid	Myristoleic acid isomer1	HMDB0002000	M2	2.46	0.0343	0.510
Glycerophosphocholine	LysoPC(O-18:1/0:0)	LMGP01060039	M1	− 0.46	0.0000	1.751
Glycerophosphocholine	LysoPC(16:0e)		M1	− 1.00	0.0000	1.678
Glycerophosphocholine	LysoPC(0:0/18:0)	HMDB0011128	M1	− 0.65	0.0000	1.591
Glycerophosphocholine	LysoPC(O-18:0/0:0)	HMDB0011149	M1	− 0.63	0.0036	1.503
Glycerophosphocholine	LysoPC(18:0/0:0)	HMDB0010384	M1	− 0.42	0.0000	1.472
Glycerophosphocholine	LysoPC(P-18:1/0:0)	HMDB0010408	M1	− 0.41	0.0021	1.465
Glycerophosphocholine	LysoPC(P-16:0/0:0)	HMDB0010407	M1	− 0.96	0.0026	1.438
Glycerophosphocholine	LysoPC(19:0)		M1	− 1.02	0.0076	1.412
Glycerophosphocholine	LysoPC(20:1/0:0)	HMDB0010391	M1	− 1.33	0.0483	1.311
Glycerophosphocholine	PC(34:2)_PC(16:0/18:2)	HMDB0007973	M3	− 0.45	0.0377	0.857
Glycerophosphocholine	PC(32:0e)_PC(32:0e)	LMGP01020029	M3	1.50	0.0480	0.812
Glycerophosphocholine	PC(37:2)_PC(37:2)mixture	HMDB0008592	M3	2.08	0.0417	0.785
Glycerophosphocholine	PC(32:0p)_PC(32:0p)	HMDB0011206	M3	1.11	0.0271	0.783
Glycerophosphocholine	PC(36:2e)_PC(36:2e)	HMDB0013418	M3	2.44	0.0413	0.774
Glycerophosphocholine	PC(36:1p)_PC(18:0p/18:1)	HMDB0008127	M3	2.44	0.0464	0.773
Glycerophosphocholine	PC(34:0p)_PC(34:0p)	HMDB0011239	M3	1.82	0.0152	0.767
Glycerophosphocholine	PC(38:6p)_PC(38:6p)	HMDB0011229	M3	− 1.62	0.0460	0.766
Glycerophosphocholine	PC(34:2p)_PC(34:2p)	HMDB0011211	M3	0.42	0.0143	0.753
Glycerophosphocholine	PC(35:2)_PC(17:0/18:2)	LMGP01011505	M3	− 2.68	0.0124	0.751
Glycerophosphocholine	PC(34:1p)_PC(34:1p)	HMDB0011210	M3	1.37	0.0153	0.750
Glycerophosphocholine	LysoPC(18:2/0:0)	HMDB0010386	M1	− 0.44	0.0292	0.743
Glycerophosphocholine	PC(33:2)_PC(33:2)	HMDB0007940	M3	− 2.06	0.0327	0.742
Glycerophosphocholine	PC(36:3)_PC(16:0/20:3)	HMDB0007980	M3	− 2.02	0.0319	0.737
Glycerophosphocholine	LysoPC(0:0/18:2)	HMDB0061700	M1	− 0.44	0.0358	0.732
Glycerophosphocholine	PC(42:3)_PC(18:1/24:2)		M3	0.18	0.0352	0.708
Glycerophosphocholine	PC(36:3)_PC(18:1/18:2)	HMDB0008105	M3	− 2.02	0.0215	0.701
Glycerophosphocholine	PC(32:1p)_PC(32:1p)		M3	− 2.43	0.0152	0.687
Glycerophosphocholine	PC(40:7p)_PC(40:7p)	HMDB0011295	M3	− 2.19	0.0271	0.687
Glycerophosphocholine	PC(33:0e)_PC(18:0e/15:0)		M3	1.27	0.0300	0.679
Glycerophosphocholine	PC(32:1e)_PC(32:1e)	HMDB0013404	M3	− 0.67	0.0124	0.650
Glycerophosphocholine	PC(35:2)_PC(35:2)		M3	0.99	0.0417	0.641
Glycerophosphocholine	PC(35:3)_PC(35:3)		M3	− 2.26	0.0157	0.576
Glycerophosphocholine	PC(30:0e)_PC(30:0e)	HMDB0013341	M3	1.32	0.0480	0.536
Glycerophosphocholine	PC(33:0p)_PC(33:0p)	HMDB0011238	M3	0.39	0.0088	0.503
Glycerophosphoethanolamine	LysoPE(0:0/22:6)	HMDB0011496	M1	− 0.76	0.0466	0.788
Glycerophosphoethanolamine	LysoPE(0:0/18:2)	HMDB0011477	M1	− 1.03	0.0271	0.748
Glycerophosphoethanolamine	PE(40:5)_PE(18:1/22:4)	HMDB0009075	M3	− 1.19	0.0239	0.690
Glycerophosphoethanolamine	LysoPE(20:4/0:0)	HMDB0011517	M1	− 1.41	0.0376	0.635
Glycerophosphoethanolamine	LysoPE(22:6/0:0)	HMDB0011526	M1	− 0.88	0.0475	0.608
Glycerophosphoethanolamine	LysoPE(18:2/0:0)	HMDB0011507	M1	− 0.84	0.0304	0.591
Micorbial metabolites	3-(4-Hydroxyphenyl)propionic acid(Desaminotyrosine)	HMDB0002199	M2	− 0.05	0.0000	19.657
Neutral glycosphingolipid	CerG3GNAc1(d34:1)_Tetrahexosylceramide(d18:1/16:0)	HMDB0004960	M3	1.29	0.0384	0.810
Neutral glycosphingolipid	CerG2(d42:1)_LacCer(d18:1/24:0)	HMDB0011595	M3	2.97	0.0343	0.762
Neutral glycosphingolipid	CerG3(d42:2)_CerG3(d18:1/24:1)	HMDB0004883	M3	3.52	0.0102	0.722
Neutral glycosphingolipid	CerG3(d42:2)_CerG3(d42:2)	HMDB0004883	M3	3.52	0.0102	0.722
Nucleoside and nucleotide	Hypoxanthine	HMDB0000157	M1	0.35	0.0000	1.587
Nucleoside and nucleotide	Xanthine	HMDB0000292	M1	− 0.03	0.0000	1.534
Nucleoside and nucleotide	Inosine	HMDB0000195	M2	0.44	0.0000	0.129
Organic acid	Glutaric acid isomers	HMDB0000661	M2	3.91	0.0384	1.107
Peptide	Gly–Val/Val–Gly	HMDB0028854/HMDB0029127	M2	3.53	0.0000	2.682
Peptide	Glp–Gly	HMDB0061890	M2	3.62	0.0000	2.348
Peptide	Phe–Phe	HMDB0013302	M2	2.02	0.0000	2.058
Peptide	Phe–Trp	HMDB0029006	M1	− 1.33	0.0000	1.868
Peptide	Ser–Hyp/Hyp–Ser	HMDB0029040/HMDB0028872	M1	− 0.35	0.0007	0.674
Peptide	Glu–Ile	HMDB0028822	M1	− 0.82	0.0088	0.635
Peptide	Glu–Gln	HMDB0028817	M2	0.67	0.0000	0.557
Peptide	Leu–Leu/Leu–Ile	HMDB0028933	M1	− 0.61	0.0000	0.367
Peptide	Ile–Val/Leu–Val	HMDB0028920/HMDB0028942	M1	− 0.53	0.0000	0.152
Peptide	Leu–Ala/Ile–Ala	HMDB0028922/HMDB0028900	M1	0.09	0.0000	0.080
Sphingolipid	Sphingosine	HMDB0000252	M1	− 1.44	0.0000	2.315
Sphingolipid	Sphinganine 1-phosphate	HMDB0001383	M1	− 1.74	0.0261	0.795
Triacylglycerol	TG(46:0)_TG(16:0/14:0/16:0)	HMDB0010411	M3	− 1.43	0.0315	2.198
Triacylglycerol	TG(48:0)_TG(16:0/16:0/16:0)	HMDB0005356	M3	− 0.85	0.0105	2.162
Triacylglycerol	TG(50:0)_TG(18:0/16:0/16:0)	HMDB0108576	M3	− 0.59	0.0075	2.091
Triacylglycerol	TG(60:9)_TG(18:0/20:4/22:5)	HMDB0045200	M3	− 0.79	0.0386	2.065
Triacylglycerol	TG(52:1)_TG(18:0/16:0/18:1)	HMDB0010431	M3	0.02	0.0075	2.059
Triacylglycerol	TG(48:1)_TG(16:0/14:0/18:1)	HMDB0010414	M3	− 1.12	0.0384	2.023
Triacylglycerol	TG(54:6)_TG(16:0/16:0/22:6)	HMDB0044613	M3	− 0.58	0.0239	1.996
Triacylglycerol	TG(54:7)_TG(18:1/14:0/22:6)	HMDB0049719	M3	0.61	0.0413	1.983
Triacylglycerol	TG(56:6)_TG(18:0/16:0/22:6)	HMDB0044747	M3	− 0.08	0.0271	1.920
Triacylglycerol	TG(52:6)_TG(14:0/16:0/22:6)	HMDB0042903	M3	0.80	0.0343	1.883
Triacylglycerol	TG(48:2)_TG(12:0/18:1/18:1)	LMGL03012670	M3	− 1.31	0.0465	1.868
Triacylglycerol	TG(48:2)_TG(16:0/14:0/18:2)	HMDB0010415	M3	− 1.31	0.0465	1.868
Triacylglycerol	TG(50:1)_TG(16:0/16:0/18:1)	HMDB0005360	M3	− 0.42	0.0157	1.841
Triacylglycerol	TG(51:1)_TG(16:0/17:0/18:1)	LMGL03010051	M3	3.30	0.0285	1.813
Triacylglycerol	TG(54:4)_TG(18:0/16:0/20:4)	HMDB0044738	M3	− 0.97	0.0102	1.801
Triacylglycerol	TG(52:2)_TG(18:0/16:0/18:2)	HMDB0044734	M3	− 0.09	0.0088	1.795
Triacylglycerol	TG(52:4)_TG(16:0/16:0/20:4)	HMDB0005363	M3	− 0.31	0.0234	1.789
Triacylglycerol	TG(54:1)_TG(18:0/18:0/18:1)	HMDB0005395	M3	2.99	0.0234	1.774
Triacylglycerol	TG(47:2)_TG(14:0/15:0/18:2)	HMDB0043227	M3	0.36	0.0413	1.733
Triacylglycerol	TG(51:0)_ TG(18:0/16:0/17:0)	HMDB0108587	M3	0.02	0.0290	1.727
Triacylglycerol	TG(49:1)_TG(15:0/16:0/18:1)	HMDB0043027	M3	− 0.54	0.0386	1.721
Triacylglycerol	TG(49:1)_TG(49:1)		M3	− 0.54	0.0386	1.721
Triacylglycerol	TG(54:2)_TG(18:0/18:0/18:2)	HMDB0005397	M3	2.83	0.0158	1.714
Triacylglycerol	TG(56:4)_TG(18:0/18:0/20:4)	HMDB0044771	M3	− 0.07	0.0300	1.699
Triacylglycerol	TG(50:2)_TG(16:0/16:0/18:2)	HMDB0005362	M3	− 1.69	0.0155	1.674
Triacylglycerol	TG(52:0)_TG(18:0/16:0/18:0)	HMDB0044722	M3	2.83	0.0124	1.653
Triacylglycerol	TG(53:1)_TG(15:0/22:0/16:1)	HMDB0043112	M3	− 0.49	0.0457	1.641
Triacylglycerol	TG(53:1)_TG(17:0/18:0/18:1)	LMGL03010119	M3	− 0.49	0.0457	1.641
Triacylglycerol	TG(56:7)_TG(16:0/18:1/22:6)	HMDB0044135	M3	− 0.72	0.0457	1.599
Triacylglycerol	TG(54:5)_TG(18:0/18:2/18:3)	HMDB0045301	M3	0.28	0.0271	1.548
Triacylglycerol	TG(54:5)_TG(16:0/18:1/20:4)	HMDB0044098	M3	0.28	0.0271	1.548
Triacylglycerol	TG(54:2)_TG(18:0/18:1/18:1)	HMDB0005403	M3	− 0.41	0.0326	1.543
Triacylglycerol	TG(54:6)_TG(16:0/16:1/22:5)	HMDB0044591	M3	0.04	0.0343	1.538
Triacylglycerol	TG(54:6)_TG(18:0/18:3/18:3)	HMDB0052887	M3	0.04	0.0343	1.538
Triacylglycerol	TG(54:6)_TG(16:0/18:1/20:5)	HMDB0044133	M3	0.04	0.0343	1.538
Triacylglycerol	TG(56:5)_TG(18:1/18:1/20:3)	HMDB0049883	M3	− 0.64	0.0498	1.440
Unannoated	X310-1-MZ310RT517		M1	− 1.13	0.0000	1.596
Unannoated	X310-2-MZ310RT526		M1	− 2.61	0.0001	1.217
Unannoated	X310-1-MZ310RT307		M1	− 1.63	0.0386	0.655
Unannoated	X310-2-MZ310RT320		M1	− 1.73	0.0416	0.539

### Metabolite enrichment and metabolic pathway analysis

**Fig. 4:**
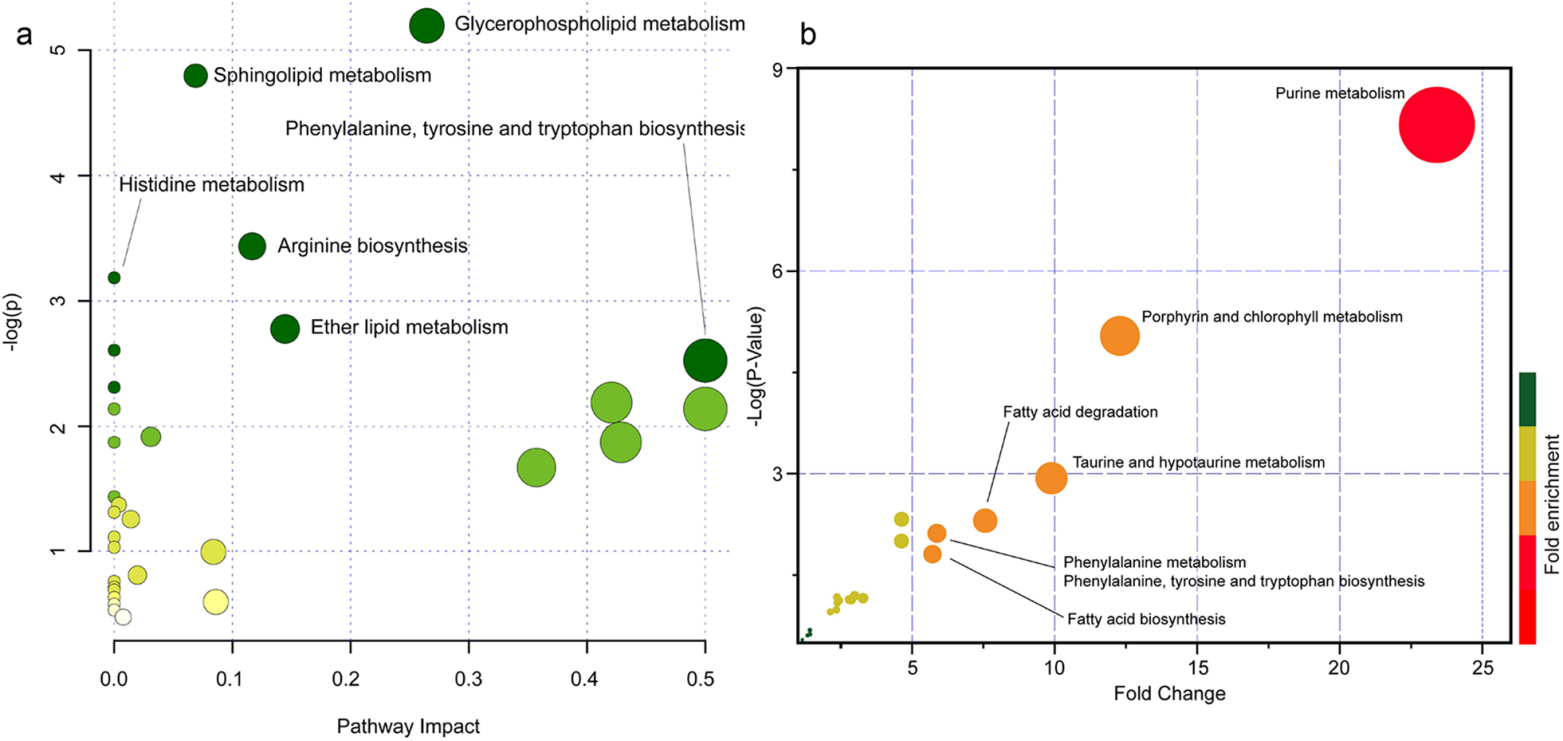
Pathway analysis of the differential metabolites between PCOS versus CON group. **a** Pathway analysis result of differential metabolites between PCOS versus control group using over-representation method in MetaboAnalyst website (*p* value < 0.05 of t-test after FDR adjusting). Hypergeometric test and relative betweeness centrality algorithm were used for pathway topology analysis, human KEGG pathway library was used. **b** Metabolites set enrichment analysis of all metabolites with HMBD identifier using quantitative enrichment analysis method. Pathway-associated metabolite sets (KEGG) containing 84 metabolite sets based on normal human metabolic pathways were used for this MSEA

Based on these identified metabolites, metabolic pathway analysis (MetPA) analysis was performed (Fig. 4a, Table 3). In Fig. 4a, − log (*p* value) and pathway impacts were the X and Y axes of the bubble diagram. It could be observed that these metabolites were significantly enriched in metabolic pathways, including glycerophospholipid metabolism, sphingolipid metabolism, phenylalanine, tyrosine and tryptophan biosynthesis, arginine biosynthesis, histidine metabolism, ether lipid metabolism. Furthermore, metabolites set enrichment analysis (MSEA) was also performed based on the Metabolites Set in the KEGG database (Fig. 4b, Table 4). The results showed that purine metabolism, porphyrin and chlorophyll metabolism, FA degradation, taurine and hypotaurine metabolism, phenylalanine metabolism, phenylalanine, tyrosine and tryptophan biosynthesis, FA biosynthesis, etc., were involved in metabolic pathways of these metabolites enriched.

**Table 3:** The pathways of the differential metabolites enriched

Pathway name	Match status	*p* value	− log (p)	Impact
Glycerophospholipid metabolism	4/36	0.00555	5.1945	0.26445
Sphingolipid metabolism	3/21	0.00826	4.7961	0.06896
Arginine biosynthesis	2/14	0.03220	3.4358	0.11675
Histidine metabolism	2/16	0.04139	3.1848	0.00000
Ether lipid metabolism	2/20	0.06227	2.7763	0.14458
Aminoacyl-tRNA biosynthesis	3/48	0.07376	2.6069	0.00000
Phenylalanine, tyrosine and tryptophan biosynthesis	1/4	0.08013	2.5241	0.50000
Linoleic acid metabolism	1/5	0.09917	2.3109	0.00000
Alanine, aspartate and glutamate metabolism	2/28	0.11195	2.1897	0.42068
Nitrogen metabolism	1/6	0.11783	2.1385	0.00000
d-Glutamine and d-glutamate metabolism	1/6	0.11783	2.1385	0.50000
Purine metabolism	3/65	0.14751	1.9139	0.03102
Ascorbate and aldarate metabolism	1/8	0.15403	1.8706	0.00000
Taurine and hypotaurine metabolism	1/8	0.15403	1.8706	0.42857
Phenylalanine metabolism	1/10	0.18879	1.6671	0.35714
Alpha-Linolenic acid metabolism	1/13	0.23835	1.4340	0.00000
Glycosylphosphatidylinositol (GPI)-anchor biosynthesis	1/14	0.25420	1.3696	0.00399
Butanoate metabolism	1/15	0.26974	1.3103	0.00000
Nicotinate and nicotinamide metabolism	1/15	0.26974	1.3103	0.00000
Glycerolipid metabolism	1/16	0.28496	1.2554	0.01402
Pantothenate and CoA biosynthesis	1/19	0.32881	1.1123	0.00000
Beta-Alanine metabolism	1/21	0.35659	1.0312	0.00000
Pyruvate metabolism	1/22	0.37005	0.9941	0.08398
Glutathione metabolism	1/28	0.44529	0.8090	0.01966
Porphyrin and chlorophyll metabolism	1/30	0.46838	0.7585	0.00000
Glyoxylate and dicarboxylate metabolism	1/32	0.49054	0.7123	0.00000
Cysteine and methionine metabolism	1/33	0.50128	0.6906	0.00000
Arachidonic acid metabolism	1/36	0.53220	0.6307	0.00000
Arginine and proline metabolism	1/38	0.55177	0.5946	0.08600
Fatty acid degradation	1/39	0.56126	0.5776	0.00000
Steroid biosynthesis	1/42	0.58856	0.5301	0.00000
Primary bile acid biosynthesis	1/46	0.62242	0.4741	0.00758

**Table 4:** MSEA pathway analysis of the differential metabolites enriched

Metabolite set	Total	Hits	FDR
Purine metabolism	65	4	0.000000
Porphyrin and chlorophyll metabolism	30	3	0.000186
Taurine and hypotaurine metabolism	8	1	0.016056
Fatty acid degradation	39	1	0.040924
Phenylalanine metabolism	10	2	0.044818
Phenylalanine, tyrosine and tryptophan biosynthesis	4	2	0.044818
Fatty acid biosynthesis	47	1	0.070722
Primary bile acid biosynthesis	46	3	0.040924
Cysteine and methionine metabolism	33	3	0.050999
Pentose and glucuronate interconversions	18	1	0.205370
Valine, leucine and isoleucine degradation	40	3	0.205370
Sphingolipid metabolism	21	5	0.205370
Glutathione metabolism	28	2	0.205370
Arginine biosynthesis	14	3	0.205370
Valine, leucine and isoleucine biosynthesis	8	4	0.208410
Arginine and proline metabolism	38	4	0.205370
Beta-Alanine metabolism	21	2	0.249570
Steroid hormone biosynthesis	85	3	0.252260
Selenocompound metabolism	20	1	0.480370
Aminoacyl-tRNA biosynthesis	48	17	0.443750
Caffeine metabolism	10	2	0.480370
Pantothenate and CoA biosynthesis	19	1	0.534600
Alanine, aspartate and glutamate metabolism	28	3	0.648530
Alpha-Linolenic acid metabolism	13	1	0.648530
Tryptophan metabolism	41	4	0.780710
Ubiquinone and other terpenoid-quinone biosynthesis	9	1	0.649670
Tyrosine metabolism	42	1	0.649670
Glycine, serine and threonine metabolism	33	5	0.863790
Glyoxylate and dicarboxylate metabolism	32	3	0.803010
Pyrimidine metabolism	39	2	0.803010
Glycerophospholipid metabolism	36	1	0.788010
Butanoate metabolism	15	1	0.803010
Pentose phosphate pathway	22	1	0.803010
d-Glutamine and d-glutamate metabolism	6	1	0.824110
Nitrogen metabolism	6	1	0.824110
Lysine degradation	25	2	0.943180
Biotin metabolism	10	1	0.869120
Nicotinate and nicotinamide metabolism	15	1	0.943180
Histidine metabolism	16	1	0.959140
Galactose metabolism	27	1	0.959140
Glycerolipid metabolism	16	1	0.959140

### ROC curves of significant metabolotics in PCOS patients and controls

**Fig. 5:**
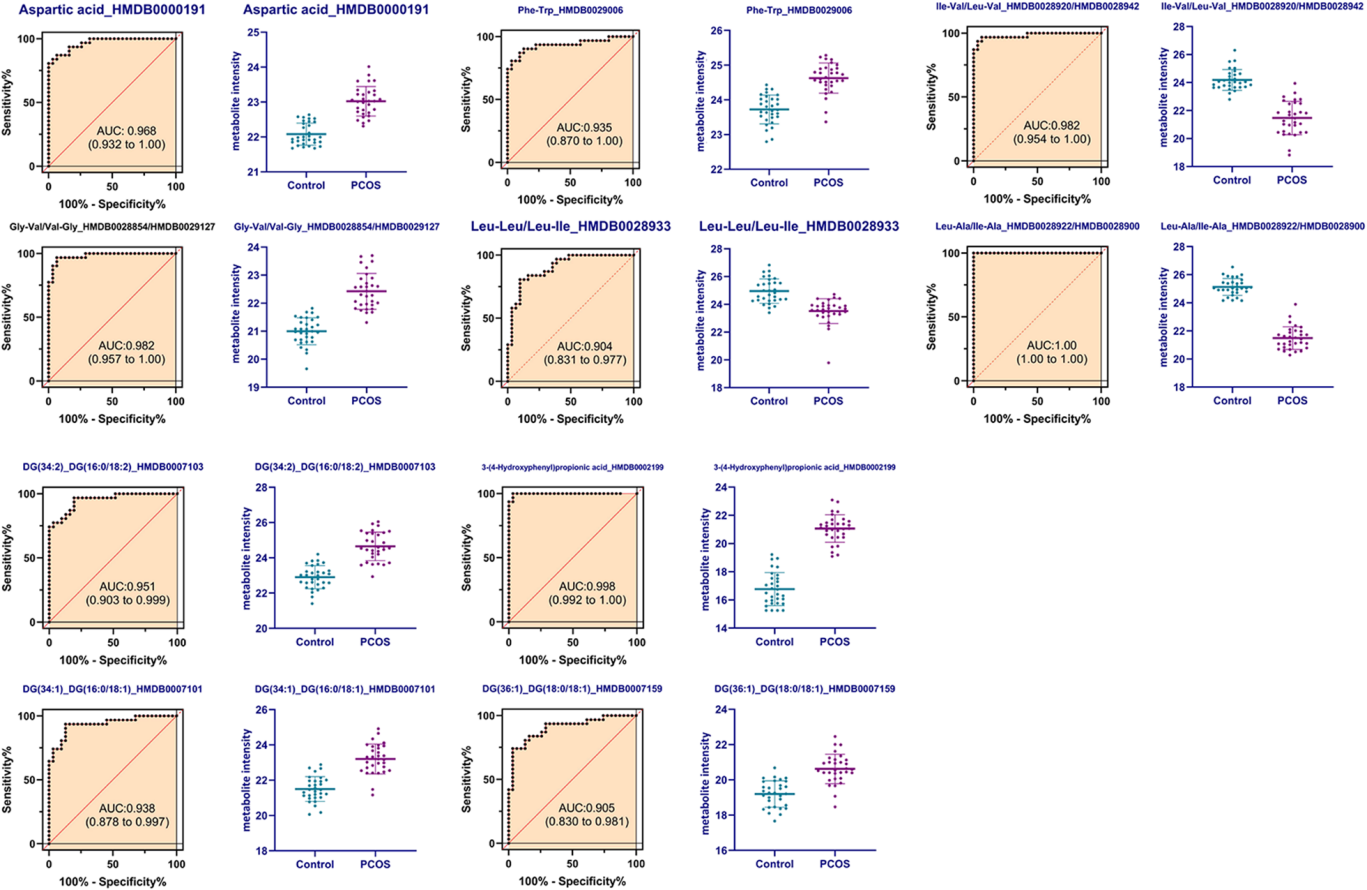
ROC-curve analysis of top 10 metabolites ranked AUC values

In order to further distinguish PCOS from controls, ROC curves analysis was also conducted on these changed metabolites. The top 10 metabolites with AUC value over 0.9 were presented in Fig. 5. These metabolites were Leu–Ala/Ile–Ala (AUC = 1.00), 3-(4-Hydroxyphenyl)propionic acid (AUC = 0.998), Ile–Val/Leu–Val (AUC = 0.982), Gly–Val/Val–Gly (AUC = 0.982), aspartic acid (AUC = 0.968), DG(34:2)_DG(16:0/18:2) (AUC = 0.951), DG(34:1)_DG(16:0/18:1) (AUC = 0.938), Phe–Trp (AUC = 0.935), DG(36:1)_DG(18:0/18:1) (AUC = 0.905), Leu–Leu/Leu–Ile (AUC = 0.904).

## Discussion

PCOS is a kind of common endocrine syndrome and a metabolic disorder, which harms the reproductive system and overall body metabolism of the patients seriously [2]. In this study, we investigated the metabolic changes in PCOS patients and healthy controls. The metabolomics analysis showed that in PCOS patients serum, there were 146 significantly changed metabolites, among them, 68 were downregulated, 78 were upregulated. These metabolites mainly belong to triacylglycerols, glycerophosphocholines, acylcarnitines, diacylglycerols, peptides, amino acids, glycerophosphoethanolamines, and FA. Pathway analysis showed that these metabolites were enriched in pathways including glycerophospholipid metabolism, FA degradation, FA biosynthesis, ether lipid metabolism, etc. Diagnosis value assessment by ROC analysis showed that AUC values of Leu–Ala/Ile–Ala, 3-(4-Hydroxyphenyl) propionic acid, Ile–Val/Leu–Val, Gly–Val/Val–Gly, aspartic acid, DG(34:2)_DG(16:0/18:2), DG(34:1)_DG(16:0/18:1), Phe–Trp, DG(36:1)_DG(18:0/18:1), Leu–Leu/Leu–Ile were all over 0.9.

Metabolomics enable to identify both the endogenous metabolites from the downstream output of the genome and the exogenous metabolites from the upstream input from the environment, therefore allowing researchers to explore the nexus of gene-environment interactions and providing unique insights into the fundamental causes of disease [10, 12]. To date, many metabolomic studies in PCOS have revealed the metabolic profiles and changes in PCOS patients under various conditions. In Zhang’s study, they recruited 286 subjects to reveal the metabolic profiling of women with HA and IR in PCOS, the identified 59 differential metabolites were related to the biosynthesis of unsaturated FAs and citrate cycle; these metabolites were meaningful to reflect the underlying mechanism of PCOS and serve as biomarkers for complementary diagnosis of HA and IR in PCOS [13]. Another study enrolled 10 PCOS patients and 10 healthy people, identified six biomarkers, L-Carnitine, LPE (22:5), Sphinganine, LPC (18:2), DHEAS and Glycocholic acid, these biomarkers belongs to metabolic pathway including lipid metabolism, carnitine metabolism, androgen metabolism, and bile acid metabolism [14]. Zhao’s metabolomics study suggested that PCOS patients and healthy control could be distinguished using a combinational biomarker of free fatty acids (FFA) 18:1/FFA 18:0, FFA 20:3, dihydrotestosterone sulfate, glycated phenylalanine, and uridine with AUC of 0.839 [15]. These studies revealed the metabolomic changes in PCOS patients, offered new insights into disease processes, but different study subjects and metabolomic techniques used impose important limitations when aiming to integrate the results of the different studies conducted to date.

In present study, over half the identified metabolites belongs to triacylglycerol (36 metabolites), glycerophosphocholine (34 metabolites), diacylglycerol (15 metabolites), and most of them were upregulated in the PCOS group. Triacylglycerol, also named triglyceride (TG), together with diacylglycerol, are the main components of lipids. As PCOS is a kind of metabolic disorders, IR and thereby induced obesity are common symptoms in PCOS patients. Hence, lipid and lipoprotein metabolic abnormalities are accompanied by the PCOS progression [16]. Previous studies also demonstrated that PCOS-associated metabolites were involved mostly in lipid metabolism [14, 15, 17]. Overweight PCOS patients usually have lipid abnormalities, including a higher level of serum TG. This was also observed in our biochemical test in Table 1, with elevated TG level in PCOS patients compared to the controls. Cross-sectional study showed that subjects with PCOS demonstrated higher waist:hip ratio, T, TG, VLDL-cholesterol concentrations (*p* < 0.05) [18]. The abnormal elevated TG level could be decreased following vitamin D supplementation for 8 weeks in PCOS women [19]. A cross-sectional study in 156 age-matched women with or without PCOS showed that diacylglycerol and triacylglycerol were inversely associated with SHBG, positively associated with homeostasis assessment of insulin resistance, free androgen index, and waist circumference [20]. This provided the evidence that specific alterations in lipid composition and function were involved in PCOS disease pathophysiology and affect PCOS clinical manifestations.

In addition, fatty acids (FAs) were also included in the identified differential metabolites in PCOS patients of this study, the three FAs (Dihomo-alpha-linolenic acid, Myristoleic acid isomer 1, Myristoleic acid isomer 2) were all downregulated in the PCOS group. Dihomo-alpha-linolenic acid is a rare polyunsaturated fatty acid (PUFA) of the ω-3 series. ω-3 PUFA supplementation has a positive effect on ovarian function and potentiates the cellular development and steroid biosynthesis in PCOS [21]. PUFA could modulate hormonal and lipid profiles of the body, lowered TG and cholesterol levels, patients with PCOS usually showed abnormal levels of PUFA metabolites. The study focused on differences in FA profiles of abdominal subcutaneous adipose tissue between pregnant women with and without PCOS found that total PUFA was lower in PCOS than non-PCOS women (*p* < 0.004) [22]. The animal model study also showed that ω-3 PUFA had an effective role in improving lipid and hormonal profile, reducing blood glucose, body weight and histopathological damages in PCOS rats [23]. Based on the positive role of FAs in normal lipid metabolism and ovarian function in PCOS, therefore, in this study, the significantly changed FAs were all down-regulated in PCOS patients, which were coincident with the previous reports.

As aforementioned, PCOS-associated metabolites were involved mostly in lipid and lipoprotein metabolic abnormalities. In the present study, pathway analysis found that these differential metabolites were associated with various pathways, especially including glycerophospholipid metabolism, sphingolipid metabolism, phenylalanine metabolism, ether lipid metabolism, purine metabolism, fatty acid degradation, fatty acid biosynthesis, etc. The untargeted metabolomics approach on PCOS follicular fluid also found significant abundance differences of glycerolipid, glycerophospholipids, sphingolipids, and carboxylic acids compared with healthy women, and these metabolism dysfunctions are contributed to declining the 2 pronuclei (PN) fertilization rate during in vitro fertilization (VIF) procedure [24]. Another LC–MS-based metabolomics showed that abnormalities of glycerophospholipid, glycerolipid, and FA metabolisms were involved in the pathogenesis of PCOS and IR complications [25]. Amino acid metabolism is also a critical metabolism pathway of the body. In this study, except for the identification of eight differential amino acids in PCOS, several related amino acid pathways were also identified, indicating the involvement of amino acid metabolism in PCOS. Zhao et al. found that, in PCOS patients, the levels of phenylalanine, tyrosine and tryptophan are generally increased, and the ovulatory dysfunction of PCOS patients was associated with raised production of serine, threonine, phenylalanine, tyrosine and ornithine [26]. Fatty acid-related pathways, including fatty acid degradation and biosynthesis were also found to be associated with the changed metabolites in PCOS of this study. And this was corresponded to the differential metabolites in PCOS compared to the healthy controls.

## Conclusion

In this study, metabolomics analysis of PCOS patients serum identified 146 significantly varied metabolites. These differential metabolites mainly belong to triacylglycerols, glycerophosphocholines, acylcarnitines, diacylglycerols, peptides, amino acids, glycerophosphoethanolamines and FA. Pathway analysis of these metabolites revealed the metabolism disorder of PCOS in lipid metabolism, including Glycerophospholipid metabolism, Fatty acid degradation/biosynthesis, Ether lipid metabolism. Leu–Ala/Ile–Ala, 3-(4-Hydroxyphenyl) propionic acid, Ile–Val/Leu–Val, Gly–Val/Val–Gly were identified as the potential biomarkers for the diagnosis of PCOS with the AUC values over 0.98, indicated a significant role of these metabolites in PCOS. Our findings suggest that the untargeted metabolomics offers a promising approach to investigate the metabolic abnormalities in PCOS patients, this may be useful for mechanism research of PCOS and provide a good prospect for PCOS diagnosis. However, our findings remain to be further investigated by large-scale metabolomics study due to the limited size of samples used in the present study.

## Data Availability

Data sharing is not applicable to this article as no datasets were generated or analysed during the current study.
